# Incorporation of Human Recombinant Tropoelastin into Silk Fibroin Membranes with the View to Repairing Bruch’s Membrane

**DOI:** 10.3390/jfb6030946

**Published:** 2015-09-16

**Authors:** Audra M. A. Shadforth, Shuko Suzuki, Raphaelle Alzonne, Grant A. Edwards, Neil A. Richardson, Traian V. Chirila, Damien G. Harkin

**Affiliations:** 1 Queensland Eye Institute, 140 Melbourne Street, South Brisbane, Queensland 4101, Australia; E-Mails: shuko.suzuki@qei.org.au (S.S); raphaelle.alzonne@supersonicimagine.com (R.A); n.richardson@qut.edu.au (N.A.R); traian.chirila@qei.org.au (T.V.C.); d.harkin@qut.edu.au (D.G.H.); 2 School of Biomedical Sciences and Institute of Health & Biomedical Innovation, Queensland University of Technology, 2 George Street, Brisbane, Queensland 4001, Australia; 3 Australian Institute for Bioengineering and Nanotechnology, University of Queensland, St Lucia, Queensland 4072, Australia; E-Mail: g.edwards1@uq.edu.au; 4 Science and Engineering Faculty, Queensland University of Technology, Brisbane, Queensland 4001, Australia; 5 Faculty of Medicine and Biomedical Sciences, University of Queensland, Herston, Queensland 4029, Australia; 6 Faculty of Science, University of Western Australia, Crawley, Western Australia 6009, Australia

**Keywords:** *Bombyx mori*, silk fibroin, tropoelastin, Bruch’s membrane, retinal pigment epithelium, age-related macular degeneration

## Abstract

*Bombyx mori* silk fibroin membranes provide a potential delivery vehicle for both cells and extracellular matrix (ECM) components into diseased or injured tissues. We have previously demonstrated the feasibility of growing retinal pigment epithelial cells (RPE) on fibroin membranes with the view to repairing the retina of patients afflicted with age-related macular degeneration (AMD). The goal of the present study was to investigate the feasibility of incorporating the ECM component elastin, in the form of human recombinant tropoelastin, into these same membranes. Two basic strategies were explored: (1) membranes prepared from blended solutions of fibroin and tropoelastin; and (2) layered constructs prepared from sequentially cast solutions of fibroin, tropoelastin, and fibroin. Optimal conditions for RPE attachment were achieved using a tropoelastin-fibroin blend ratio of 10 to 90 parts by weight. Retention of tropoelastin within the blend and layered constructs was confirmed by immunolabelling and Fourier-transform infrared spectroscopy (FTIR). In the layered constructs, the bulk of tropoelastin was apparently absorbed into the initially cast fibroin layer. Blend membranes displayed higher elastic modulus, percentage elongation, and tensile strength (*p < 0.01*) when compared to the layered constructs. RPE cell response to fibroin membranes was not affected by the presence of tropoelastin. These findings support the potential use of fibroin membranes for the co-delivery of RPE cells and tropoelastin.

## 1. Introduction

While strategies for tissue regeneration are often based upon the replacement of lost cells, such efforts often ignore the significant contribution of extracellular matrix (ECM) components to tissue structure and function. A good example of this problem is illustrated through the attempts to treat age-related macular degeneration (AMD) of the retina. In short, although the pathology of AMD involves significant changes to both cellular and ECM components, most efforts to date have been largely focused on replacing only the cellular components and, especially, retinal pigment epithelial (RPE) cells [1,2,3,4]. In doing so, healthy RPE cells are ultimately delivered into sites containing an abnormal composition and arrangement of ECM components. In order to address this issue, a number of groups have explored the potential of a variety of biomaterials as temporary ECM substitutes to support the RPE cells during cultivation and implantation [5,6]. In our case, we have focused on the development of a substitute prepared from the silk structural protein, fibroin [7,8]. Using this strategy, we have demonstrated the feasibility of establishing functional monolayers of RPE cells grown on fibroin membranes. These RPE monolayers share several important features with those found within the healthy retina, including apical-basal polarity, patterns of growth factor secretion and phagocytic function [9]. As such, fibroin membranes have potential as a vehicle for implanting cultured RPE cells into AMD patients. Since the fibroin membranes will eventually degrade, the incorporation of ECM components, or their precursors, within the fabricated membranes may further facilitate subsequent development of a more permanent ECM. The aim of the present study, therefore, was to examine the feasibility of incorporating ECM components found naturally within the outer retina. More specifically, we have examined the incorporation of the precursor protein from which elastin fibres are produced, tropoelastin [10].

Our focus on tropoelastin arises from considering the composition of the ECM that resides immediately posterior to the RPE, a structure known as Bruch’s membrane. A functional, native Bruch’s membrane contains an elastin fibre-rich core that is thought to facilitate tissue compliance during cycles of tissue expansion and recoil as blood flows through the adjacent capillaries of the choriocapillaris [11]. The elastic properties of Bruch’s membrane may also serve to protect the delicate connections that exist between RPE cells and the adjacent photoreceptor cells [12]. However, age-related changes, such as the accumulation of abnormal deposits referred to as drusen, disrupt the biochemical and mechanical properties of Bruch’s membrane [11]. Moreover, an aged Bruch’s membrane deters the survival of both endogenous, as well as implanted, RPE cells [13,14,15,16,17]. Importantly, RPE cells have been shown to produce microfibrils, and lysyl oxidase, the enzyme responsible for converting tropoelastin into elastin fibres [18]. Thus, by implanting RPE cells in conjunction with tropoelastin it may be possible to regenerate the core element of a functional, native Bruch’s membrane following degradation of the fibroin-based delivery template. In addition, since tropoelastin shares similar elastic properties with elastin [10], it may also be possible to create fibroin-tropoelastin constructs with physical and mechanical properties that are more favourable for establishing and implanting RPE cell cultures than constructs based solely on fibroin.

Two strategies for incorporating tropoelastin into fibroin membranes were examined in this study. Membranes were produced from fibroin solutions supplemented with recombinant human tropoelastin (fibroin-tropoelastin blend) or prepared from alternating cast solutions of fibroin and tropoelastin (layered approach). In the case of the blend, we commenced by optimizing the amount of tropoelastin that can be added to fibroin solution without negatively impacting on the attachment of RPE cells to the resulting membranes. Freestanding membranes were subsequently produced from the optimal blend formulation, and by using the layered approach. The two types of biomaterial membrane were subsequently compared in parallel with standard fibroin membranes using a variety of criteria, including morphology (scanning electron microscopy), secondary structure (Fourier-transform infrared spectroscopy-attenuated total reflectance, FTIR-ATR), the distribution of tropoelastin (immunofluorescence), the cultivation of RPE cells, and mechanical properties. These studies led to some unexpected findings, especially in regard to how tropoelastin in solution interacts with cast fibroin membranes.

## 2. Results and Discussion

### 2.1. Properties of Fibroin and Tropoelastin Solutions

**Figure 1:**
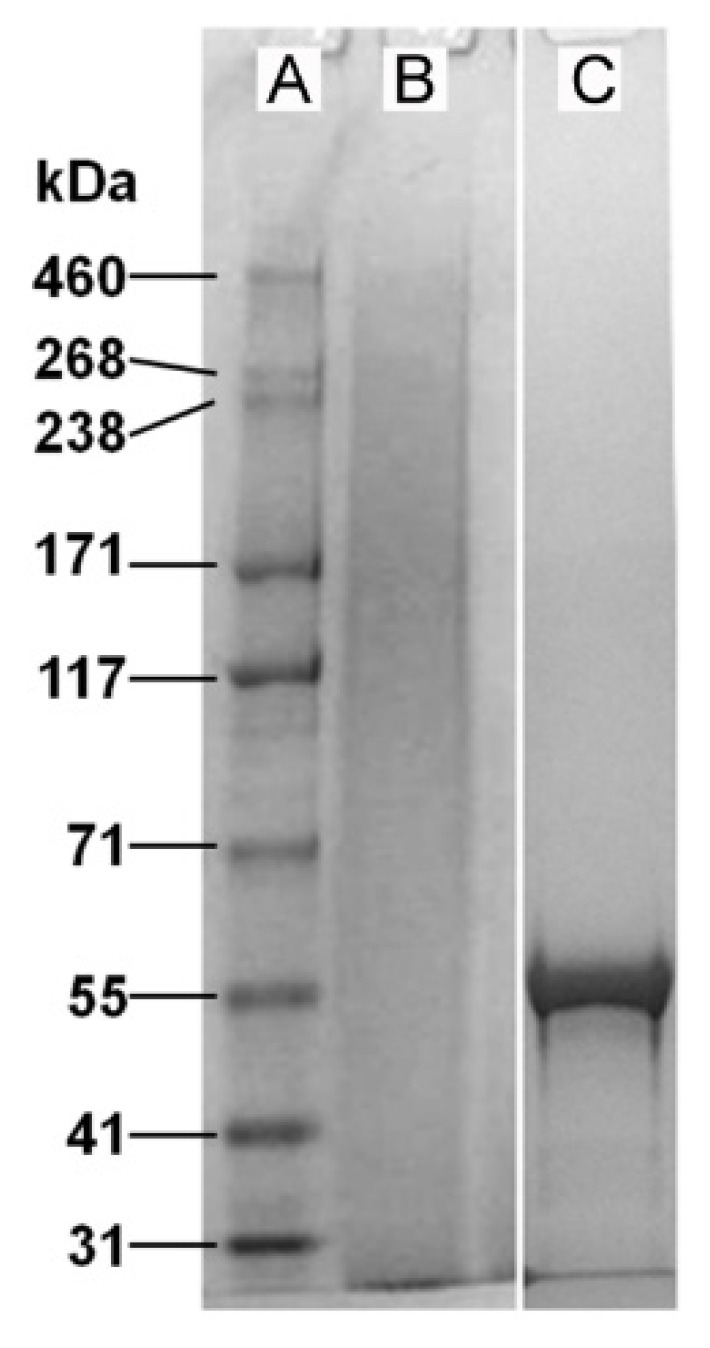
Relative molecular weight distribution for purified native *Bombyx mori* silk fibroin (**B**) and recombinant human tropoelastin (**C**), as displayed by gel electrophoresis. While the extracted fibroin proteins present as a broad range of peptide fragments, recombinant human tropoelastin has a defined molecular weight of approximately 55 kDa. The left lane (**A**) shows a selection of molecular weight markers.

During their extraction from silkworm cocoons [19], a significant proportion of the native fibroin proteins (heavy chain 350 kDa and light chain 26 kDa) were cleaved into fragments of varying molecular weights (Figure 1). In contrast, human tropoelastin produced via recombinant DNA technology [20] displayed a single band by gel electrophoresis, at approximately 55 kDa (Figure 1). The aqueous solutions of fibroin and tropoelastin mixed readily with increasing ratios of up to 50% tropoelastin by weight. Phase separation was observed when combining solutions at 10% tropoelastin by weight (resulting in a cloudy solution); however, the resulting dried films were transparent and smooth when cast in plastic (polystyrene) tissue culture dishes. 

### 2.2. Effect of Tropoelastin on RPE Cell Attachment to Fibroin

**Figure 2:**
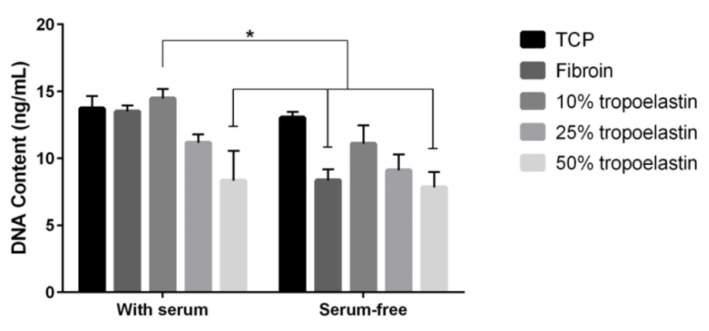
Comparison of cell attachment of retinal pigment epithelial cell line (ARPE-19) on tissue culture plastic (TCP) coated with either fibroin solution, or fibroin mixed with increasing concentrations of tropoelastin (proteins blended in solution before coating TCP). Evidence of cell attachment was examined after 4 h in either the presence or absence of 10% (v/v) fetal bovine serum (with washing prior to measurement). Each substrate was tested in triplicate. Bars represent mean values ± standard error of the mean from three experiments. The difference between fibroin with 10% tropoelastin used in the presence of serum and the other identified bars was statistically significant (*p* < 0.05).

Since fibroin supports the attachment and growth of RPE cells [8] and tropoelastin has also been shown to positively influence cell attachment [21,22], we examined different blend ratios of fibroin and tropoelastin with the goal of identifying an optimal formulation for the resulting blend membrane. As demonstrated in Figure 2, a consistent trend was observed towards an optimal RPE cell attachment (as defined by DNA content), in either the presence or absence of serum (10% v/v), using a tropoelastin-fibroin ratio of 10 to 90 parts by weight. This result was consistent with prior reports [21,22] and has been explained as the optimal ratio between the two proteins.

### 2.3. Gross Morphology of the Freestanding Membranes

**Figure 3:**
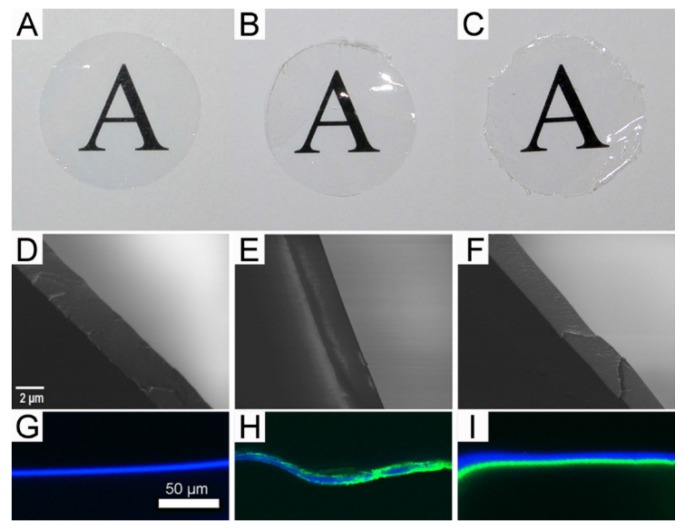
Physical appearance of membranes prepared from either fibroin alone (**A**, **D,** and **G**), tropoelastin-fibroin blend (10:90 ratio) (**B**, **E,** and **H**), and layered solutions of fibroin and tropoelastin (**C**, **F,** and **I**); (**A**–**C**): gross appearance of each membrane when placed over printed text (16-mm diameter discs); (**D**–**F**): internal structures revealed by scanning electron microscopy following freeze-fracture; and (**G**–**I**) visualization of tropoelastin (green) by immunolabelling and confocal fluorescence microscopy (the presence of fibroin revealed as blue autofluorescence).

Having established the optimal blend ratio of fibroin to tropoelastin for RPE cells, we proceeded to test the feasibility of producing freestanding membranes from the optimal blend, as well as layered constructs produced by sequential addition and drying/stabilization of aqueous solutions containing each protein (fibroin followed by tropoelastin, then fibroin again). Both types of membrane were prepared in glass Petri dishes coated with Topas^®^ polymer as described previously [23]. In brief, the Topas^®^ coating facilitated the subsequent removal of fibroin-based membranes from the glass Petri dishes and was itself delaminated easily, leaving behind the protein membranes. The membranes produced from the optimal blend (Figure 3B) or by layering (Figure 3C) were physically comparable to the standard fibroin membranes produced routinely in our laboratory (Figure 3A). All membranes were transparent and could be cut into the 16-mm diameter discs required for our custom-designed Teflon^®^ cell culture chambers [8]. Nevertheless, the layered membranes (Figure 3C) were noticeably more brittle during excision, resulting in discs with uneven edges (Figure 3C). While no layers were evident within the membranes examined by scanning electron microscopy (SEM) following freeze fracture (Figure 3 D–F), a distinct band of positive immunolabelling for tropoelastin was observed within the layered construct by confocal fluorescence microscopy (Figure 3I). In contrast, an uneven distribution of staining for tropoelastin was observed within the blend membrane (Figure 3H). Unexpectedly, only a single band of fibroin autofluorescence was observed within the layered constructs (Figure 3I). This result initially suggested to us that perhaps one of the fibroin layers had detached during handling, but repeated attempts using multiple samples revealed the same result. Moreover, no evidence of a detached fibroin sheet was observed in any sample mounted for confocal microscopy. We, therefore, embarked upon an FTIR analysis of the layered composites to determine the fate of the apparently “missing” third layer.

### 2.4. Analysis of Membrane Structure by Fourier-Transform Infrared Spectroscopy-Attenuated Total Reflectance, “FTIR-ATR”

**Figure 4:**
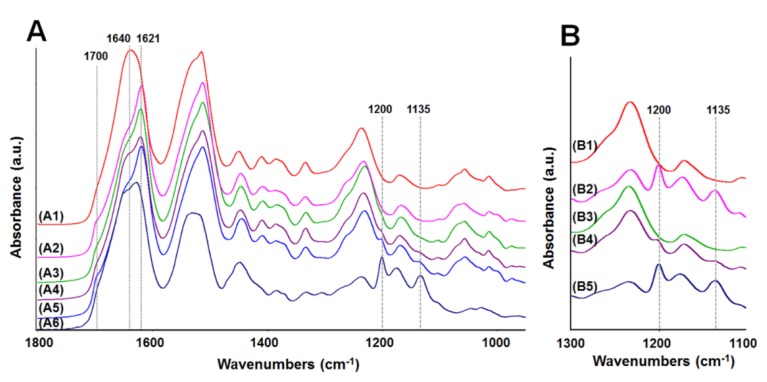
FTIR-ATR spectra of membranes. (**A**) (in the range of 1800–950 cm^−1^): (A1) fibroin membrane (water annealed at 25 °C, 6 h), (A2) fibroin membrane (water annealed at 60 °C, 12 h), (A3) blend membrane (fibroin:tropoelastin = 90:10), (A4) three-layered membrane—side 1, (A5) three-layered membrane—side 2, (A6) tropoelastin membrane (untreated); and (**B**) (in the range of 1300–1100 cm^−1^): (B1) two-layered membrane (untreated)—fibroin side, (B2) two-layered membrane (untreated)—tropoelastin side, (B3) two-layered membrane (methanol treated)—fibroin side, (B4) two-layered membrane (methanol treated)—tropoelastin side, (B5) tropoelastin membrane (untreated).

The FTIR-ATR spectra in the range of 1800–950 cm^−1^ were used to examine the surface structure of the different biomaterial membranes (Figure 4A). The amide I region between 1720 and 1580 cm^−1^ is traditionally used for analysis of the secondary structure in proteins, and this region has been well described for silk fibroin [24]. In the spectrum of the standard fibroin membrane (water-annealed for 6 h at 25 °C) (Figure 4A, A1), both the amide I band shape and its peak maximum at 1640 cm^−1^ indicate a significant amount of random coil component. The fibroin (Figure 4A, A2) and blend (Figure 4A, A3), membranes that were water annealed for 12 h at 60 °C, revealed a strong band at 1621 cm^−1^ and a shoulder at 1700 cm^−1^, corresponding to β-sheet structures and their aggregates [24]. If the layered (fibroin-tropoelastin-fibroin) membrane truly had three layers as expected, both surface spectra should reveal a similar fibroin signature. One side of the layered membrane (Figure 4A, A4) did reveal a fibroin signature similar to those described above; however, the other side (Figure 4A, A5) revealed more pronounced β-sheet bands. This may be a result of the additional methanol treatment of the initial fibroin layer after tropoelastin was added. The other side (Figure 4A, A5) also revealed two weak bands at 1200 and 1135 cm^−1^ (indicative of tropoelastin) (Figure 4A, A6), suggesting that one side of the layered membrane consists of a mixture of fibroin and tropoelastin near the surface. The possibility that the methanol treatment might be removing some of the tropoelastin layer was also considered. The tropoelastin bands were used to investigate the stability of tropoelastin in two-layered (fibroin-tropoelastin) membranes before and after methanol treatment (Figure 4B). The spectrum for the tropoelastin side before methanol treatment (Figure 4B, B2) presented two bands at 1200 and 1135 cm^−1^ which correspond to the spectrum of the untreated tropoelastin membrane (Figure 4B, B5). After methanol treatment these tropoelastin bands had dramatically decreased (Figure 4B, B4). Indeed, a thin membrane (thickness of ~1 µm) of tropoelastin was readily soluble in pure methanol which was demonstrated in a separate investigation to confirm FTIR results. 

**Figure 5:**
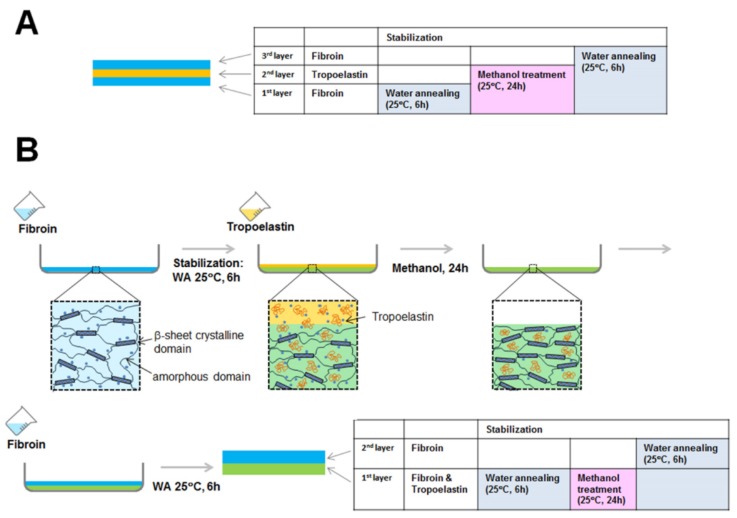
Schematic scenario of the predicted (**A**) and actual (**B**) outcomes achieved during the creation of a layered membrane of fibroin and tropoelastin. Based upon FTIR-ATR data, we propose that the bulk of applied tropoelastin is absorbed and subsequently trapped within the initially created fibroin membrane. Therefore, only two main layers are detected by immunofluorescence/microscopy.

In considering the differences in relative molecular weight distributions for fibroin and tropoelastin (Figure 1) and our previous studies of fibroin membrane permeability (≤70 kDa using FITC-dextran) [25], the following explanation for the “missing layer” was devised (Figure 5). When tropoelastin solution was cast onto the first fibroin layer it is proposed that some tropoelastin penetrated through the loosely stabilized fibroin hydrogel network. These tropoelastin molecules were subsequently trapped within the fibroin network by drying and treatment with methanol. Hence, the first fibroin layer had a well-distributed content of tropoelastin, as demonstrated by immunofluorescence. A small proportion of tropoelastin remaining on top of the first fibroin layer is also likely to have been washed away by methanol treatment. The final layer applied (second fibroin layer) would then appear as a single blue layer by autofluorescence.

### 2.5. Cytocompatibility of the Membranes

**Figure 6:**
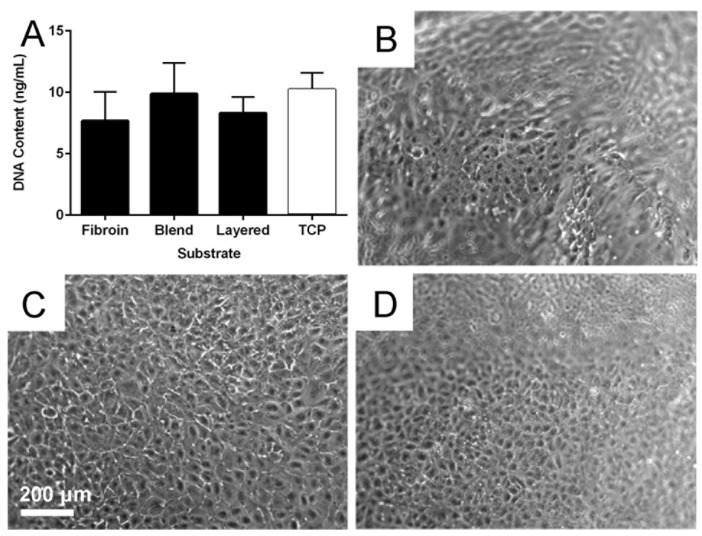
Retinal pigment epithelial (RPE) cell behaviour on biomaterial membranes. Quantification of RPE cell numbers (**A**) using the PicoGreen^®^ assay after 3 days culture on fibroin, blend, and layered membranes; Tissue culture plastic (TCP) was included as control substrate. Phase contrast micrographs of RPE cells after 21 days of growth on fibroin (**B**); blend (**C**); and layered (**D**) membranes. The undulating nature of the suspended membranes is the reason some areas of panels (B) and (D) are out of focus. The scale bar represents 200 µm and applied to the micrographs.

The cytocompatibility of the fibroin, blend and layered membranes was examined over an extended culture period using current best practice culture conditions [26]. An assessment of cell numbers after three days’ culture (Figure 6A) was quantified using the PicoGreen^®^ assay (DNA content provides an indication of cell numbers). There was no statistically significant difference in the number of cells attached across the three biomaterial membrane types, and when compared to the TCP control substrate. The RPE cells seeded on each membrane type showed a similar appropriate morphology over the extended culture period (Figure 6B–D).

### 2.6. Mechanical Properties of the Membranes

**Figure 7:**
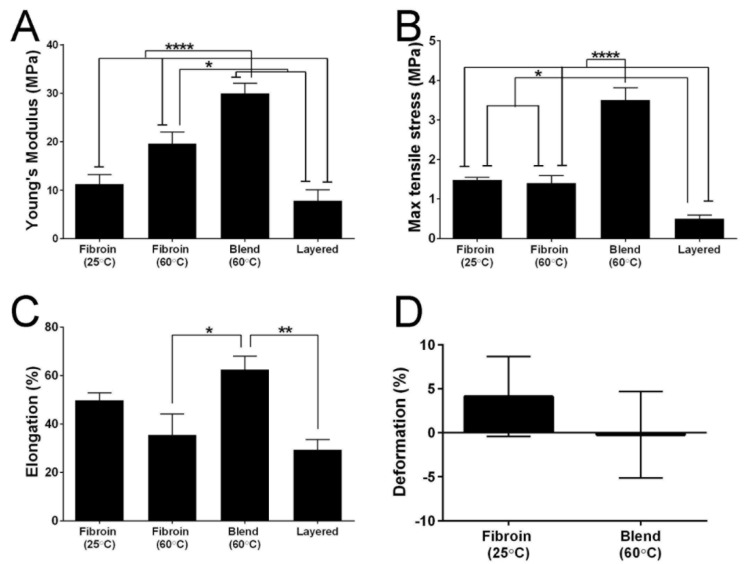
Quantitative comparison of the tensile properties of biomaterial membranes. (**A**) Young’s modulus; (**B**) maximum tensile strength; (**C**) elongation to break; and (**D**) deformation/recoil capacity after 200 cycles. Bars represent mean values ± standard error of the mean. Asterisks indicate differences are statistically significant (* *p* < 0.05, ** *p* < 0.01, **** *p* < 0.0001).

While our primary goal is to use fibroin as a delivery vehicle for tropoelastin, it is possible that combining the two proteins may produce changes in mechanical properties that impact upon their handling during RPE cell culture and surgical implantation. As a consequence, we compared the mechanical properties of standard fibroin membranes to those displayed by the blend and layered constructs. The results (Figure 7) revealed significant differences between the membranes. The layered membranes, while considerably thicker than the other membranes (data presented as mean values ± standard error of the mean; layered membranes 16.667 ± 0.639 µm, compared to fibroin membranes water-annealed at 25 °C 3.610 ± 0.369 µm, fibroin membranes water-annealed at 60 °C 4.612 ± 0.540 µm, and blend membranes 6.112 ± 0.362 µm), were also more brittle (Figure 7B). In contrast, membranes prepared using a 10% tropoelastin by weight blend with fibroin were the stiffest (Figure 7A), however, they were also strong (Figure 7B) and elastic (Figure 7C). The most interesting results were seen in the standard fibroin membranes that were water-annealed at 25 °C. There was no statistical difference between these membranes and the fibroin membranes water-annealed at 60 °C, however, they did show different properties. The former were the only membranes that had a Young’s modulus (Figure 7A) within the range of native Bruch’s membrane (7–19 MPa; [27]) and a useful combination of maximum tensile strength (Figure 7B) and elongation properties. This is especially clear when considering there was no difference in elongation at break when compared to the blend membrane (Figure 7C). There was also no statistical difference in recoil capacity of the fibroin (water-annealed at 25 °C) and blend membranes after 200 cycles (Figure 7D) of stretching. 

## 3. Experimental Section 

### 3.1. Production of Aqueous Solutions of Fibroin

The procedure has been previously described in detail by our group [28]. Briefly, dried *Bombyx mori* silkworm cocoons (Tajima Shoji Co. Ltd., Yokohama, Japan) were boiled in a solution of sodium carbonate containing 0.85 g of salt for each gram of cocoon material. This procedure removed the sericin outer coat from the core fibroin protein. The resulting fibrous material was washed and dried, and then dissolved (at 60 °C for 4 h) in a concentrated solution of lithium bromide (9.3 M) to obtain a silk concentration of approximately 10% wt./vol. The fibroin solution was subsequently filtered using syringe filters in succession with pore size 0.7 µm and 0.2 µm. This step is performed slowly to avoid shearing forces that could promote spontaneous gelation. The filtrate was dialyzed against water using a dialysis cassette with a molecular mass cut-off of 3.5 kDa (Slide-A-Lyzer, Pierce Biotechnology) using six changes of water over three days. The resulting fibroin solution was filtered again as above and used to produce fibroin membranes.

### 3.2. Preparation of Films Cast in TCP Wells: Fibroin and Tropoelastin Solutions Blended in Different Ratios

Films of fibroin and tropoelastin were prepared by the method reported by [21] with some modifications. Briefly, tropoelastin (freeze-dried powder) was dissolved in cold MilliQ water (4 °C) to make the concentration of 1.78%, and kept in an ice bath for 2–3 h with occasional vortex mixing. The low temperature is required to prevent coacervation of the solution (self-aggregation of hydrophobic domains). The tropoelastin solution was slowly added to a cold fibroin solution (1.78%) by a pipet, and mixed by inverting the tube slowly. The volume ratio of the fibroin solution to the tropoelastin solution was mixed over the range 90:10, 75:25, and 50:50. The mixture solutions were cast into wells of 24-well TCP plates and dried in a fan-driven oven for 12 h at room temperature. For structural stabilization of fibroin with tropoelastin, β-sheet formation was induced by water annealing the plates in a vacuum oven at 60 °C, −80 kPa with ~100 mL water in a beaker, for 12 h, followed by drying in a fan-driven oven for 12 h at room temperature.

### 3.3. Cell Culture of the Human RPE Cell Line ARPE-19

ARPE-19 cells were routinely cultured using the Miller’s medium formulation [29]; minimum essential medium, alpha modification (MEM-α, M-4526) supplemented with N1 supplement (N-6530), glutamine-penicillin-streptomycin (G-1146), non-essential amino acids (M-7145), taurine (T-0625), hydrocortisone (H-0396), and triiodo-thyronin (T-5516). All of these components were purchased from Sigma Aldrich. This medium formulation allows RPE cultures to be incubated at 37 °C using a standard level of 5% CO_2_ air. Cultures were established in the presence of 10% fetal bovine serum, and after 24 h this serum level was decreased to 1%. Stock cultures were fed two to three times per week, and passaged routinely using Versene (15040-066, Life Technologies, Carlsbad, NM, USA) and TrypLE™ (12563-011, Life Technologies), between passages number 23 and 28. An independent STR profile analysis of our working stocks by the Garvan Institute of Medical Research revealed a 100% match with reference ARPE-19 cell line CRL-2302.

### 3.4. Testing the Attachment of RPE Cells on Films of Fibroin and Tropoelastin Blended in Different Ratios

The cell attachment was quantified on films prepared by blending solutions of fibroin and tropoelastin (Section 3.2). RPE cells (ARPE-19) were seeded at a density of 40,000 cells/cm^2^ and incubated at 37 °C, 5% CO_2_ for 4 h using Miller’s medium without serum. Fibroin films and TCP (used with and without serum) were used as control substrates for ARPE-19 cell attachment. Each substrate type was tested in triplicate, with the experiment performed in triplicate and quantified using the Quant-iT PicoGreen^®^ dsDNA kit (Molecular Probes™, Life Technologies).

### 3.5. Preparation of Fibroin Membranes

Fibroin membranes were cast using a custom-made casting table as described previously by our group [25]. The thickness of fibroin membranes was measured using an upright micrometer and only areas of membrane 3 µm ± 1 µm thick were used. For structural stabilisation of fibroin membranes, β-sheet formation was induced by the water-annealing of the membranes in a vacuum oven at −80 kPa with ~100 mL water (beaker) for 6 h at room temperature (25 °C). The permeability of fibroin membranes has been previously examined using a horizontal diffusion cell using three model molecules [25].

### 3.6. Preparation of Freestanding Membranes of Fibroin and Tropoelastin, Proteins Blended in 90:10 Solution Ratio

Freestanding membranes of fibroin and tropoelastin blend were prepared by the method outlined above (Section 3.2), except that only the 90:10 volume ratio of the fibroin solution to the tropoelastin solution was used. For casting, 45-mm Petri dishes were first coated with a Topas^®^ (a commercial hydrophobic cyclic olefin copolymer) film (1 mL of a 7% solution) by the evaporation from a solution in cyclohexane. The Topas^®^ solution formed a hydrophobic film on the glass, facilitating easy removal of the membranes from the dishes later. The mixture solution (1.78%, 1 mL) was poured into the dish, and dried in a fan-driven oven for 12 h at room temperature. For structural stabilisation of fibroin with tropoelastin, the blend membranes were water annealed using a vacuum oven with a container of water and kept at −80 kPa at 60 °C for 12 h, followed by drying in a fan-driven oven for 12 h at room temperature. The membranes were peeled from the Topas^®^ film and used for cell culture and mechanical testing. The thickness of the membranes used was 2–3 µm.

### 3.7. Preparation of Freestanding Layered (Fibroin-Tropoelastin-Fibroin) Membranes

Layered membranes were fabricated using separate aqueous solutions of fibroin and tropoelastin, layered in sequence and followed by stabilisation after each layer. Before casting any protein solutions, 45-mm Petri dishes were first coated with a Topas^®^ film. The layered membrane was prepared as following. Firstly, 1 mL of 0.59% fibroin solution was cast and dried in a fan-driven oven at room temperature overnight, followed by water annealing in a vacuum oven with a beaker of water at −80 kPa at room temperature, for 6 h. Then 1 mL of 0.59% tropoelastin solution was cast and dried at 4 °C for four days. The tropoelastin layer was stabilized by treatment with methanol (5 mL) for 24 h at room temperature. Finally, 1 mL of 0.59% fibroin solution was cast on top of the tropoelastin layer, and stabilized by water annealing as above. The volumes used were calculated to generate 1 µm-thick layers of each protein, which would result in a 3 µm-thick layered membrane. A 1 µm-thick membrane of tropoelastin was cast and was not treated with methanol (untreated) as a comparison for FTIR-ATR studies. 

### 3.8. Suspension of the Membranes in Custom-Made Teflon^®^ Chambers

Discs (16-mm diameter) of biomaterial membrane were inserted into custom-made chambers designed by our group, which are manufactured from interlocking Telfon^®^ rings specifically for cell culture use [8]. The combined membrane and chamber were sterilised together by immersion in 70% ethanol for 1 h at room temperature, air-dried, and washed thoroughly with phosphate-buffered solution (PBS). The custom-made chamber suspends the biomaterial membrane (reminiscent of the commercially available Transwell^®^ insert system) creating an apical compartment (upper chamber) and a basal compartment (lower chamber) on either side of the membrane. This culture setup is required for the development of a polarised epithelial culture.

### 3.9. Visualization of Tropoelastin within the Membranes Using Immunofluorescence

Samples of fibroin, blend, and layered membranes were incubated with a primary monoclonal antibody to tropoelastin (BA4, 1:50, ab21599, Abcam, Cambridge, UK). The secondary antibody used was an Alexa 488-conjugated goat-anti-mouse IgG (Molecular Probes^®^, Life Technologies). Negative controls for immunostaining were incubated with the secondary antibody only. Confocal laser scanning microscopy (Nikon A1R, Nikon Corporation, Tokyo, Japan) was used to image immunofluorescence.

### 3.10. Testing Cell Growth of RPE Cells on the Membranes

Cell growth after 72 h on the fibroin, blend, and layered membranes was compared and quantified. RPE (ARPE-19) cells were seeded (4000 cells/cm^2^) on discs (6-mm diameter) of the different biomaterial membranes and evaluated for total cell numbers, 72 h after seeding using the Quant-iT PicoGreen^®^ dsDNA kit (Molecular Probes™, Life Technologies). This experiment was performed using discs of the freestanding biomaterial membranes held down by rubber o-rings in the wells of 96-well plates.

### 3.11. Extended Culture of RPE Cells on the Membranes

RPE (ARPE-19) cells were seeded (10,000 cells/cm^2^) onto the apical surface of biomaterial membranes suspended in Teflon^®^ chambers (Section 3.8). All membrane types; fibroin, blend, and layered membranes, were precoated with a commercial Collagen I solution obtained from porcine origin (0.3 mg/mL, Cellmatrix^®^, Nitta Gelatin Inc., Osaka, Japan) diluted in MilliQ water. Cultures were incubated at 37 °C and 5% CO_2_, and culture media was changed twice weekly. Phase contrast light microscopy was used to examine the cultures over a two month culture period.

### 3.12. Fourier-Transform Infrared Spectroscopy of the Membranes

The FTIR-ATR spectra of the membranes (fibroin, blend, and layered) and tropoelastin were collected using a Nicolet FTIR spectrometer (Thermo Electron Corp, Waltham, MA, USA), equipped with a Nicolet Smart Endurance diamond ATR accessory. Each spectrum was obtained by co-adding 64 scans over the range of 4000 to 525 cm^−1^ at a resolution of 8 cm^−1^. The OMNIC 7 software package (Thermo Electron Corp, Waltham, MA, USA) was used to analyse and plot the spectra.

### 3.13. Mechanical Testing of the Membranes

Strips (1 cm × 3 cm) were cut from each membrane type and subjected to tensile measurements in an Instron 5848 micrometer, equipped with a 5 N load cell and a set gauge distance of 14 mm. The membranes were mounted in pneumatic grips and submersed in PBS at 37 °C in a BioPuls™ unit for 5 min prior to testing. Stress-strain plots were recorded, and the Young’s moduli were computed in the linear region. The mean values were calculated from results generated by 4–6 measurements for each specimen. In addition, cyclic tensile loading/unloading testing was carried out to evaluate recovery behaviour. The testing experiments were set up as above. However, the following method profile was used: the repeated cyclic loading/unloading was performed at strain of 20% in the stress-strain curve, which is the linear region, with ± 5% strain of loading/unloading and the rate of 14 mm/min. The number of cycles performed was 200 cycles. Four measurements were performed for each specimen. From stress-strain plots, the areas under the curve of cycles 10 and 200 were calculated and used to evaluate deformation using the following equation:

Deformation (%) = ((Area _cycle 10_ − Area _cycle__200_)/Area _cycle__10_) × 100

### 3.14. Statistical Analyses

Results from cell attachment and growth assays were analysed for statistical significance using a two-way ANOVA followed by a Tukey’s multiple comparisons test (with the two variables being either “substrate and serum”, or “substrate and time”). Mechanical testing data were analysed using a one-way ANOVA with Tukey’s test comparing membrane types (with the variable being “membrane type”). Recoil testing data for fibroin and blend membranes were analysed using an unpaired *t* test (since comparing only two independent samples). All statistical analyses were performed using GraphPad Prism, V 6. 

## 4. General Discussion

AMD is a leading cause of permanent vision loss in the elderly. Significant efforts are therefore underway in countries with ageing populations to address this disease. Consideration of the underlying histopathology indicates that therapies based upon the replacement of both cellular (e.g., RPE cells), as well as extracellular tissue components, may well be required. To this end, we have previously demonstrated that freestanding membranes prepared from silk fibroin provide a potential vehicle for delivering RPE cells into the subretinal space [8,9]. The present study builds upon this research by examining the feasibility of incorporating elastin (in the form of tropoelastin) into these same fibroin membranes. In doing so, we have proposed that fibroin membranes may provide a vehicle for co-delivering RPE cells and tropoelastin to the subretinal space. Moreover, since tropoelastin displays similar elastic properties to elastin, we considered that the mechanical properties of fibroin membranes may be significantly altered when combined with tropoelastin.

With regard to our first aim, our data confirms the feasibility of incorporating human recombinant tropoelastin into fibroin membranes. Varying results, however, are achieved according to the methods used. In short, membranes prepared from blended solutions of the two proteins displayed a more heterogeneous composition than those produced using a sequential layering method. We propose that the patchy distribution most likely results from either phase separation or specific molecular interactions between the two proteins when present together in solution. By comparison, our subsequent analyses by FTIR suggest that the more homogenous distribution of tropoelastin achieved using the layering approach is due to absorption and subsequent fixation of this protein within the originally cast fibroin membrane (by treatment with methanol). This result suggests that membranes prepared via the absorption/fixation method should theoretically support a more even profile of tropoelastin delivery following implantation to the subretinal space. Nevertheless, the choice of technique is also likely to be influenced by consideration of membrane mechanical properties.

Our study of the effects of tropoelastin on the mechanical properties of fibroin membranes, when blended, led to some unexpected results. While others have reported reduced stiffness of fibroin membranes following inclusion of tropoelastin [21,22], we have presently reported the opposite result. On the surface, this conflicting data seems quite difficult to resolve. A close comparison of the methods used, however, reveals several significant variations including the source of cocoons, fibroin isolation protocol, water annealing temperature and the thickness of membranes used. In our experience, any one of the parameters alone can have significant effects on the properties of fibroin membranes. Thus, in combination, the differing processes could well have been responsible for the variations in response to the tropoelastin observed between each study.

A comparison of blended *versus* “layered” strategies for incorporating fibroin and tropoelastin is also an interesting exercise. On the basis of their superior strength and elasticity, it could be concluded that the blended membranes are superior to the more brittle “layered” constructs. Nevertheless, a revised formulation whereby the tropoelastin is simply absorbed and trapped, without an additional fibroin layer being deposited, is worthy of investigation. In any case, the key comparison to make is how closely each membrane resembles the mechanical properties of Bruch’s membrane. It is, thus, significant that fibroin membranes water-annealed at 25 °C and membranes prepared using the layered approach are closest to native Bruch’s membrane in terms of Young’s modulus [27]. Therefore, on this basis, and in combination with the more uniform distribution of tropoelastin, we propose that the layered membranes are at present the better option to pursue in order to address both issues of ECM delivery, as well as matching the desired mechanical properties.

## 5. Conclusions

Reconstructing both the cellular and ECM components of diseased and injured tissues is an important area of tissue engineering and regenerative medicine. The incorporation of a tropoelastin component in fibroin membranes, while maybe not bestowing benefits to mechanical properties, offers a potential vehicle for the delivery of RPE cells and Bruch’s membrane ECM components into the subretinal environment of patients with AMD. Future studies will need to investigate the suitability of these membranes in a pre-clinical animal model.
